# Implementing Information Resources to Support Shared Decisions in Australian Primary Care: A Qualitative Perspective of an Antimicrobial Stewardship Strategy

**DOI:** 10.3390/antibiotics15020216

**Published:** 2026-02-17

**Authors:** Ruby Biezen, Kaleswari Somasundaram, Stephen Ciavarella, Tim Monaghan, Kirsty Buising, Jo-Anne Manski-Nankervis

**Affiliations:** 1 Department of General Practice and Primary Care, The University of Melbourne, Melbourne, VIC 3004, Australia; 2 National Centre for Antimicrobial Stewardship, Department of Infectious Diseases, The University of Melbourne, Melbourne, VIC 3004, Australia; 3 The Guidance Group, Royal Melbourne Hospital, Melbourne, VIC 3000, Australia

**Keywords:** patient information resources, co-design, decision support, primary care, general practitioners, antibiotics, disease management, primary care providers, consumers, patients

## Abstract

Background/Objectives: Inappropriate use of antibiotics contributes to the rise in antibiotic-resistant bacteria and can result in adverse drug effects for individuals. Informed discussions between patients and general practitioners (GPs) can help ensure that treatment decisions about antibiotic use align with the best health outcomes for individuals. Methods: We implemented a set of information resources designed to support clinical discussions and decision-making for patients with common infections in primary care. A suite of patient information sheets, which had been co-designed with primary care providers and consumers, were implemented in eight general practices in metropolitan Melbourne and regional Victoria, from August to November 2020. Results: Post-implementation evaluation, conducted through interviews with 15 primary care providers and 13 patients, revealed that the information sheets were simple, easy to use and generated discussion within consultations. GPs reported using the sheets to reinforce their decision-making during consultations with patients, reduce potential conflict, provide alternatives to antibiotic prescriptions, and offer patients a written summary of management recommendations. Patients found the sheets informative and that they made it easier to understand their diagnosis and to manage their conditions. Both GPs and patients agreed that the content was relevant and effectively enhanced patients’ knowledge of disease conditions, treatment options, and when to seek medical advice and were facilitators of meaningful conversations during consultations. Conclusion: These resources are acceptable in Australian primary care and publicly available for use by GPs, pharmacists and patients in Australia.

## 1. Introduction

Antibiotic resistance poses a significant and growing threat to global health, endangering the effectiveness of treatments for various infectious diseases. The misuse of antibiotics contributes to the development of antibiotic resistance in bacterial pathogens and can lead to significant adverse drug reactions, posing both immediate and long-term risks to human health [1,2]. Many common infections such as upper respiratory tract infections do not always warrant antibiotic use. Research indicates that inappropriate antibiotic use may stem from diagnostic uncertainty, physicians’ beliefs that patients are more satisfied when prescribed antibiotics, assumptions that patients expect antibiotics and patients’ demand for these medications [3,4,5,6,7,8,9,10,11]. The Clinical Care Standards for antimicrobial stewardship, introduced by the Australian Commission on Safety and Quality in Health Care [12], emphasize that patients should be provided with comprehensible information about their clinical condition, its natural progression, and the available treatment options. This ensures that patients can make informed decisions about their healthcare.

Currently, there are very few decision aids for antibiotic use available in Australia that are aimed at patients [13], and there is a lack of a documented and transparent process that involves both healthcare providers and consumers in their development. Furthermore, very few have undergone formal evaluations to assess their acceptability in clinical settings.

In 2019, our team co-designed and developed shared decision support information resources to promote antimicrobial stewardship for patients with primary care providers (general practitioners (GPs), pharmacists, and practice nurses) and consumers [14]. These tools included seven information resources (acute cystitis, cellulitis, bronchitis, leg ulcers, otitis media, sinusitis and tonsillitis) to support clinical discussion and decision-making for patients with common infections in primary care and to help empower patients to better understand their illness and facilitate self-management for conditions that may not need antibiotics [15].

The aim of the study was to evaluate the usability and acceptability of the seven patient information sheets in general practices in Victoria, Australia, from the perspective of primary care providers and patients.

## 2. Results

### 2.1. Participants

Between October 2020 and March 2021, 15 primary care providers (14 GPs and one practice nurse) and 13 patients were interviewed. In the primary carer providers group, 53% (n = 8) were female, with the majority aged between 31 and 40 years of age (n = 10, 67%). Eight practices were from regional and rural areas. In the patient group, 53% (n = 8) were female, with the majority recruited from metropolitan practices (n = 9, 60%). Patients were recruited from all age groups (please see Tables S1 and S2 in the Supplementary Material). During Victoria’s COVID-19 lockdown and restrictions (July to October 2020), five patients were recruited via participating GPs at their practices. Following the easing of restrictions in late 2020, an additional eight patients were recruited from practice waiting rooms. Patients who did not view or receive patient information resources during their GP consultations were provided with copies via email and later asked to reflect on them during the interviews.

### 2.2. Interview Findings

During the intervention period (3 August to 30 November 2020), there were 494 community information sheet page views and 407 unique page views of the individual patient information sheets recorded (please see Supplementary Material S3) [15].

Five main themes were identified from the 28 qualitative interviews:

1. Driver for use of information sheets;
2. Content and design;
3. Usability (barriers to using information sheets);
4. Communication and engagement;
5. Delivery and access.

#### 2.2.1. Drivers for Use of Information Sheets

GPs and patients cited different reasons for utilizing these resources. GPs primarily used the information sheets to reinforce their decision-making during consultations, as an alternative to prescribing antibiotics, and to provide patients with a written summary of management recommendations. Patients who engaged with these information sheets during their consultations reported that the sheets enhanced their understanding of their treatment and management options for their condition.

The information sheets served as a valuable written summary or advice, especially for patients who struggled to recall information discussed during their consultations. This aided patients in self-management of their conditions.

*“I believe, as far as I’ve understood from patients during the years that I’ve practiced, most of the time they cannot remember the whole information that has been given to them, so having something written always helps.”*

GP9, female, age 41–50.

*“… I think that what’s good about it is that it does point to what she can do to feel better… this is how you get better from a sore throat. Rather than just get out of my room, you don’t need it [antibiotic prescription].”*

GP15, female, age 31–40.

GPs indicated that the information sheets were effective in reinforcing their guidance on the appropriate use of antibiotics, especially in situations where antibiotics were not necessary.

*“It was nice I think, because a lot of the things that I say anyways was just reiterated by them [patient information sheet], and so if they read it again to say, “Oh yeah, this is what my GP said.”. Anyways, it just helps reinforce the point that we’re trying to make….”*

GP14, male, age 31–40.

Nearly all patients emphasized the importance of the information sheets for providing knowledge about their diagnosis and guiding them on how to manage their conditions independently.

*“Yes, it… helped me understand it a bit more as I had no clue what tonsillitis does to you or that it was that easy to spread as well. It was very helpful.”*

Patient 3, male, age 18–25.

*“Well, it would help me to understand my symptoms and what to do, how to care for myself, and what to expect. Because I suffer with a bit of anxiety, and I would need as much information about my diagnosis. It’s very important, yes.”*

Patient 9, female, age 56–65.

#### 2.2.2. Content and Design

Participants noted that the patient information sheets were well-designed, informative, visually appealing, and easy to understand. The content was concise yet comprehensive, enabling patients to evaluate their condition and determine if a visit to their doctor was necessary.

*“Well, I find it very easy [to read]. I like the colours, I think colour is important in capturing peoples’ attention and I like the design, and I like that it’s sort of, kind of cute with little cartoons and I think I’m a very visual person, so visually it appeals to me.”*

Patient 1, female, age 46–55.

*“Yeah, the content’s easy to understand, especially for a plumber like myself… not good with words but it was very easy to understand and just breaks down what I need to know about it…”*

Patient 3, male, age 18–25.

*“…it tells you what is actually happening in your body. A lot of people get frightened if they start coughing up mucus or stuff like that… I can look at this and say, “well okay I’ve got this, this, this and that’s happening”. So yeah, I can look at the ‘what can I do to feel better’... All the basic information is there…”*

Patient 8, female, age 65+.

Participants also highlighted the importance of design features like bold headings, a straightforward question-and-answer format, simple sentence structures, eye-catching illustrations, and meaningful images. These features were seen as important elements for effectively delivering messages to the public.

*“What does it feel like? Feel, look, help, prevent, when should I see a doctor? It is five really easy questions, and the information is super simple. There are no overly complicated sentence structures. The key points of medications and symptoms are bolded. From a graphical representation point of view, I think they are about as simple as you could get without taking away from the main message. I think they are really well designed.”*

Patient 5, male, age 36–45.

*“The little animated little pictures in there were very helpful. I had a read through all of them, I printed out all the cases that you had for it and I stuck it on my monitor so it would remind me of when to give it out.. In general, I thought the information sheets were really well written; plain language, very good…Well, look, they’re beautiful. Have that on record, they’re beautiful pictures.”*

GP14, male, age 31–40.

Participants also emphasized the importance of incorporating colour and graphic representations.

*“… it’s got bright colours and the headings [that] attract you… you’ve explained what that picture says, you explain ‘what does it feel like’ and it explains ‘what can I do’, you put the picture there with tea and the honey and the lemon and your antibiotics and the little child or person coughing into their arm and the doctor there. So, to me it’s the perfect little sheet.”*

Patient 8, female, age 65+.

*“I think the illustrations are kind of—it’s very eye-catching, it’s very clear. I quite like the question/answer kind of format. Yeah, it’s just got that simplicity about it, it’s not overwhelming to a patient to have that...”*

GP13, female, age 51–60.

*“There wasn’t much of it that I didn’t like. I thought they were very eye-catching, I thought they were simple format, easy to read and I did show a few different people… not just nurses or doctors, but different professions here. They all said the same thing, aesthetically it was very good and the information they thought it was yeah, very easy to follow, quick and easy to read and we gave them information that they wanted. And I said about the pharmacy thinking it was great. They said, ’Oh can we put these up?’”*

Practice nurse 1, female, age 25–30.

Suggestions were made to optimize the content, particularly emphasizing education about the natural progression of the disease. These suggestions highlighted that antibiotics are not always necessary for a perforated tympanic membrane in the context of otitis media and that the presence of fever does not automatically indicate the need for antibiotics.

#### 2.2.3. Usability

Patients appreciated that the information sheets provided useful information about infectious diseases, management and treatment options, all without the use of complex medical terminology.

*“I think it’s good because it doesn’t have a huge amount of writing, so it’s just brief and to the point, because if it’s too detailed you don’t read it… And not, necessarily, in my case because I can understand this, but I think the fact that it’s written quite simply, and not using big medical words, is good for the average person to understand.”*

Patient 13, female, age 26–35.

The patient information sheets were seen as valuable resources to be utilized before, during, and after a GP consultation. Patient interviews highlighted their significance in helping individuals determine when to visit a GP, facilitating discussions with their doctors about treatment and management options during the consultation, and providing a take-home resource for further reading and reference.

*“… it would be good to get it before—I know it’s good that obviously the doctor gives it to you but to have it out there so… it’s a reference before as okay this is what I need… it does say at the end ‘do I need to see a doctor.’”*

Patient 2, male, age 31–40.

*“I think it would be more helpful for the doctor to provide rather than prior, because otherwise you are just trying to identify things.. what does it feel like? I have got this, I have got that, and then it might not be that at all. It could be something completely different.”*

Patient 5, male, age 41–50.

Some GPs believed that the information sheets would be most effective when used during the consultation. They felt that this timing would allow them to reinforce their guidance on antibiotic use and better educate patients on disease management.

*“Yes, you tell them while discussing the ‘why you probably don’t need antibiotics and when you would’—while I’m talking about that I pull it up… Remembering that they’re there…”*

GP15, female, age 31–40.

*“I would see it, I can guess that at the end of the consult, really as a kind of supporting piece of information. So, I hope that I would have covered that in the consultation but, you know, for instance I might forget to say what can you do to stop it spreading. It also gives you that tick of closure, so right, we’ve talked about that, hopefully we’ve covered the points from here just to reiterate the points in an info sheet.”*

GP13, female, age 51–60.

The intervention period during the COVID-19 pandemic was a barrier in this study. Many face-to-face general practice consultations became telehealth consultations, and the majority of patients with respiratory tract infection symptoms were referred to respiratory clinics rather than being seen in a general practice. As a result, the patient information sheets were not utilized as frequently as anticipated.

*“So pretty limited in COVID context…A lot of the information sheets are infective conditions and unfortunately with our health practice set up a lot of people that had any infective symptoms including fever were seen in the carpark where we were consulting with full PPE (Personal Protective Equipment) and you were going out to the carpark... trying to minimise you coming back and forth and just taking what you need there. So, unless for whatever reason there was a suggestion of what the patient was coming with you weren’t taking these information sheets out to the carpark.”*

GP12, female, age 31–40.

Some GPs noted that patients lacked technological proficiency, making it challenging to deliver the information sheets via email or direct them to online resources.

*“Now, we found it difficult… because we’ve got a very elderly population who aren’t particularly internet-savvy, we haven’t been able to use some of the telehealth, video-type consultations. Internet has been a bit of a barrier as well with certain people, so we’ve tended to use telephone over the video because of that. And so, in saying that, it’s been hard to use those sorts of leaflets in that area.”*

GP5, male, age 41–50.

Some GPs expressed that remembering to distribute the information sheets was a challenge due to their busy routines in general practice.

*“I have indeed had some patients [would benefit from these leaflets] but unfortunately it wasn’t in my sort of immediate thought process…”*

GP12, female, age 31–40.

*“So, I’m thinking from the GP’s point of view, it’s kind of like oh no, not another thing I have to remember. I’d love it if I was to type in the word bronchitis there was a pop up, something which it would then—it would just say ‘print this off for the patient’, or something.”*

GP10, female, age 61–70.

While most GPs preferred the information sheets in colour, their clinical settings were limited to black and white printing. They felt that black and white prints were less appealing to patients. Additionally, some practices were transitioning away from using paper altogether.

*“I actually liked the colour ones better. So, the hard copies were actually able to come in colour, and that was heaps better, because they just look nicer…So, I ran out of a number of them quicker. There were a few conditions that you see more frequently, so having that as a handout was so great. And then you print it black and white, it just didn’t look as pretty, like colour.”*

GP11, female, age 41–50.

*“But these, kind of are a bit—it takes me back so I’m trying to go away from paper. The fact this that these are colourful, I don’t have a colour printer so they’re going to come out black and white, they’re not going to look nearly as attractive, et cetera. And I’m trying to move away from bits of paper.”*

GP13, female, age 51–60.

#### 2.2.4. Communication and Engagement

Participants noted that the information sheets were written at an appropriate level and effectively enhanced their understanding of the condition.

*“I think it’s written in plain English, which is good. What does it feel like? …painful burning or a stinging sensation when urinating, and the need to urinate more often. Yeah, I think short sentences [are good] …”*

Patient 13, female, age 26–35.

*“So last week a patient with cellulitis, and they were saying, “Well, how can I get it? How can it actually be?” So it just helps to be able to give them a picture type of thing with that.”*

GP7, male, age 41–50.

The information sheets served as an acceptable communication tool to help discussion with patients regarding appropriate use of antibiotics and to reduce potential conflicts about advice that may arise when patients indicated that they required antibiotics but GPs assessed that antibiotics were not necessary for their condition.

*“It’s primarily been most useful for tonsillitis I would say—which is the area where people are most expecting antibiotics…”*

GP15, female, age 31–40.

*“So, antibiotics are not usually needed, but the patient will say, “Well when will I need one?” And you say, “Well this, this and this.” “Well, I don’t want to come back and see you, so can you give me a script?”*

GP3, male, age 61–70.

*“Yeah. Well, for tonsillitis a few patients that have come to me in the past and had antibiotics, you know, but the guidelines haven’t really recommended antibiotics for it, and I sort of just used it as a bit of a template to run through. And it’s pretty good. You’ve just got the section; will antibiotics help and sort of just more through that...”*

GP6, male, age 31–40.

Although the patient information sheets were not utilized as planned during the pilot phase due to the pandemic, those interviewed believed that these sheets would help educate patients about disease management, treatment options, prevention, and antibiotic use. They also thought the sheets would encourage patients to consult their GPs if symptoms did not improve or worsened.

*“… and the bit down here is very good about what you can and can’t do, like covering your mouth, washing your hands, getting rid of the tissues and all that sort of stuff. It’s a really good information... And the fact that you don’t really—well you might not usually need to see your doctor but if it gets any worse yes, definitely go and see him. I think that that’s a really good helpful hint.”*

Patient 8, female, age 65+.

#### 2.2.5. Delivery and Access

Participants were divided on the preferred mode of delivery for the patient information sheets. Some favoured online access, citing the convenience and the challenges of printouts during telehealth consultations as reasons. The desire for ‘paperless’ options, especially during the COVID-19 pandemic, when concerns about handling physical materials were heightened, also influenced their preference. Conversely, others preferred printouts, particularly older patients without online access; those who found it easier to have a physical copy on hand; and those who preferred not to search online. Overall, offering both online access and printed copies were seen as the acceptable delivery options moving forward.

*“I would have probably preferred that it had been emailed to me…Because I’ve got email on every device I have ever owned and lying in bed at night with my phone, I could have a quick read. So, it’s quite accessible that way...”*

Patient 1, female, age 46–55.

*“I think the main thing is going to be with telehealth going forward is how can we integrate this make it a little bit more user-friendly to the GP to be able to give out electronically.”*

GP2, male, age 31–40.

*“I will appreciate if he gave me a printout, because I’ve got it in front of me rather than go looking for it and get lost and on Google or whatever it is.... If he gives it to me well, I’ve got it. Yeah, I’m quite happy.”*

Patient 12, male, age 65+.

*“For me, I just ask the patient what they want, and they usually will tell me. My default is the paper based one, because when you give them something physical it’s a little bit more…present in their mind, rather than nebulous email type thing. Sometimes I’ll do both, so I’ll give them it, and then I’ll say, ‘I’ll email you as well just so you have a copy, because it’s pretty useful.’ … I think moving forward I probably want to do both.”*

GP14, male, age 31–40.

*“Middle ear infections… I’d really like to have a pad of those to give out. And your bronchitis, because they’re more your—that elderly ones as well. And sometimes your younger, chesty, yeah kind of ones….The tonsillitis, the middle ear and the bronchitis are probably the ones that I would use, because they’re probably the ones that, by the time when I am seeing them, it’s usually earlier in the stage of their infections, and most of them would very much benefit from a handout like that…”*

GP11, female, age 41–50.

Most participants suggested pharmacies and practice waiting rooms were also considered as ideal locations for accessing or distributing these information sheets.

*“… and they (patient information sheets) might be really handy going to chemists, those ones. That’s where I can see the UTI ones being with the pharmacists giving them out… because that’s often where they do go there first up.”*

GP1, male, age 41–50.

*“Yeah, because I know past, not recently, but in the past, some of the pharmacies that I’ve gone to they would have like a stand, like a bookshelf. It holds fliers in it and you’d have all the different illnesses or sicknesses like asthma, sore back or whatever. Maybe something like that in a pharmacy, yeah.”*

Patient 10, male, age 56–65.

Some participants suggested that the information sheets could be displayed as posters in waiting rooms.

*“If I didn’t come in for it I, probably, wouldn’t have gone and picked it out and looked at it, but if it was on the wall, like a poster type thing, I would probably look at it and read it because there’s not much to do in the waiting room.”*

Patient 13, female, age 26–35.

*“But I like that it’s—if you blew it up a bit bigger you could then just have it as a poster around the waiting room, which is also good. So, in terms of the size and that kind of thing, that’s what I would do.”*

Practice Nurse 1, female, age 25–30.

One patient suggested that the various information sheets could be compiled into a comprehensive booklet.

*“Do you know what I really think, if and I mean I know it probably would be costly, but if you could print up something like that in maybe a booklet or something with all of these little cases if you have on this whole thing then perhaps even think about sending it out to households.”*

Patient 8, female, age 65+.

## 3. Discussion

Our study evaluated the acceptability and usability of seven patient information resources in eight metropolitan Melbourne and regional general practices in Victoria, Australia. The result supported the acceptance of these information sheets through using a co-design process to include end-users’ voices in the development. This qualitative study found that these resources were acceptable to both primary care providers and patients. Participants highlighted that these tools improved patients’ knowledge of disease and conditions and their treatment and management options, including appropriateness of antibiotic use, and that they supported clinical decisions. Ultimately, users felt that they would help reduce inappropriate antibiotic prescribing and use, thereby resulting in better patient outcomes in primary care.

These resources were developed with the aim of providing information that primary care providers and consumers deemed important when addressing patients’ needs, managing common infections and encouraging communication between patients and their primary care providers [14]. During the co-design phase, we ensured that end-users provided input and feedback around content and design, access and delivery mode. Results from this study demonstrated the importance of the co-design phase, where participants agreed that the information resources were visually appealing and captured the audience and written to a standard that is easy to understand, while relaying important messages for patients managing their infective symptoms.

GPs and patients perceived their use of information resources differently; our study found that GPs believed these resources were needed to reinforce their clinical decisions, offer written advice or a summary of the discussion, and as an alternative to prescribing antibiotics. On the other hand, patients found the information sheets acceptable and useful as they enhanced their knowledge and understanding of their condition(s). Regardless of how they viewed the use of these tools, it encouraged communication and reduced potential conflicts between GPs and patients. Previous studies have shown that shared decision-making tools encourage communication between GPs and patients [16,17] and facilitate discussion about antibiotic expectations, including benefits and harms, to assist patients in making an informed decision. Even when used infrequently [18], the sheets were still deemed as useful to patients, who felt valued in the decision-making process for their disease management.

Effective communication around the need for antibiotics may reduce potential conflict. A UK study conducted to understand GPs’ perception around negotiating antibiotic prescribing decisions with patients found that optimizing antibiotic prescribing created tensions for GPs, resulting in potential conflicts during consultation [19]. Recommendations included the need for resources such as visual tools to support prescribing decisions, especially in cases when antibiotics were not needed or prescribed. Interventions such as decision aids have been shown to reduce conflict and increase patient knowledge around awareness and management of their illness, resulting in the reduction in antibiotic prescribing [17,20,21,22]. Our study found that communication using the resources increased discussion around appropriate antibiotic prescribing.

Our study found that participants expressed a desire for the information resources to be available before, during, and after GP consultations. Patients mentioned they would access these information resources to help them understand their illness symptoms and, in particular, when to see their GPs. Providing patients with evidence-based information may help to better focus the discussion between patients and their GPs during their consultation. Patients who did not receive the information resources during a consultation mentioned that they would have found them helpful during their GP visit.

Both GPs and patients valued the options to access patient information sheets in both online and hard-copy formats. Online access would suit patients who prefer not to have printouts and can access information electronically, while patients with limited or no online access would prefer printed copies. In addition, the study highlighted that the mode of delivery should be tailored to the primary care setting, such as general practice waiting rooms, pharmacies, and online government websites. Having access to these information resources available through general practices and pharmacies may encourage consistent messaging as well as collaboration between these settings within primary care.

The systematic design of the study using a co-design phase [14] and the subsequent evaluation of the information resources were a strength in this study. The co-design process allowed participants to prioritize their health needs and to develop a product that met the needs of both health professionals and patients. The resulting evaluation phase reiterated the importance of these information resources and their acceptance of using these tools to increase patient knowledge of disease management, as well as increase communication and reduce potential conflict between GPs and patients. While the study was based in Victoria, Australia, we were able to include a range of participants from different demographics including location (metropolitan and regional), gender, age, level of education, and years of experience (GPs only).

The small number of participants recruited in Victoria means that the results may not be generalizable across Australia. However, the purpose of the qualitative study was not to achieve generalisable results but to ensure that the information resources, developed using the co-designed phase, met the real-world needs of GPs and patients. In addition, the COVID-19 pandemic significantly hindered the use of the patient information sheets during the intervention period. Telehealth consultations were introduced, with a resultant decline in face-to-face consultations [23], reducing the opportunities to provide the information resources as a handout to patients. In particular, Victoria, Australia, was subject to multiple lockdowns during 2020–2022, minimizing transmission of not only SARS-CoV-2 but also many respiratory diseases, therefore reducing the need for the respiratory infection information sheets. Nonetheless, the strong relationships the researchers had with GPs in the study allowed the study to adapt to the needs of the GPs and their patients, providing links for GPs and patients to access the information resources when needed and posting printouts to practices for practice staff to send to patients where appropriate. These resources are publicly available for use by GPs, pharmacists and patients (please see Supplementary Material S3) [15].

## 4. Materials and Methods

### 4.1. Study Design

It was a qualitative study of GPs and patients attending general practices who were recruited to utilize seven patient information sheets (via printed copies, files saved locally on the GPs’ general practice computers, and National Centre for Antimicrobial Stewardship (NCAS) community page website) over a four-month period (3 August–30 November 2020). Participating GPs and practices nurses were asked to utilize these patient information sheets developed in 2019–2020 using a co-design process [14] with patients presenting with a respiratory tract infection (acute bronchitis, tonsillitis, otitis media, and rhinosinusitis), skin and soft tissue infections (cellulitis and leg ulcers), or urinary tract infections (cystitis) where appropriate.

### 4.2. Recruitment

An email was sent to 120 VicREN (Victorian Primary Care Practice-based Research and Education Network) practices to advertise the project. Phone calls were made to 10 practices who expressed an interest in the research project. Of those, eight Victorian practices (four metropolitan and four regional practices) consented to participate in the study. Zoom conference calls and phone calls were made to participating practices to discuss the study in detail and to answer any questions the practice participants might have.

Two primary care providers from each of the eight participating practices were invited to participate in an interview during the intervention period to discuss acceptability and usability of the information tools. In addition, it was planned that up to four patients from each practice who attended the practice with symptoms of respiratory tract infection, skin and soft tissue infection, or urinary tract infections were to be approached to be invited to participate in an interview in the practice waiting room. However, this was not possible during the pandemic, especially when most face-to-face consultations were replaced by telehealth consultations. GPs who used the tool with their patients were asked to assist with patient interview recruitment by providing them with researchers’ contact details if they were interested in participating in the interviews. After the lockdown period ended at the end of October 2020, a member of the study team attended participating practices’ waiting rooms to recruit patients. Those interested were screened for eligibility if they were coming in for an infective illness. Primary care providers were provided with a $100 e-gift card for the one-hour interview, and patients were provided with a $50 Coles e-gift card for a 30 min interview.

### 4.3. Data Collection and Analysis

Interviews were conducted via phone or Zoom videoconference as per participants’ preference between October 2020 and March 2021. All interviews were digitally recorded and transcribed verbatim.

Qualitative data were analyzed using a thematic approach. The coding scheme was developed by grouping recurrent ideas during data analysis and then refined under themes and subthemes. Data were coded independently by two researchers with differences in perspective negotiated with a third researcher until consensus was reached. Data were managed using NVivo 12 (QSR International)

The number of page views of the patient information sheets stored on the NCAS website was recorded.

### 4.4. Ethics

Ethics approval was obtained from The University of Melbourne Human Ethics Advisory Group (Ethics ID: 1954925.2).

## 5. Conclusions

Although the COVID-19 pandemic limited their use, interviews with primary care providers and patients indicated that the information sheets were well-received in both the metropolitan and regional areas of Victoria. Participants expressed satisfaction with the information sheets and were willing to continue using them where appropriate. By providing information on the symptoms, treatment, and management of common infections—such as acute bronchitis, middle ear infections, nose and sinus infections, sore throat, urinary tract infections, cellulitis, and leg ulcers—these sheets educated patients on the appropriate use of antibiotics, improved their understanding of disease symptoms and management, encouraged communication between patients and GPs, and supported clinical discussions and decision-making for patients with common infections in primary care. These information resources will be embedded within a broader antimicrobial stewardship primary care intervention in the near future.

## Data Availability

Data can be made available upon reasonable request.

## Supplementary Materials

The following supporting information can be downloaded at: https://www.mdpi.com/article/10.3390/antibiotics15020216/s1, Table S1: Characteristics of primary care providers; Table S2: Characteristics of patients. S3: Shared decision support information resource.

## References

[1] World Bank (2016). Drug-Resistant Infections: A Threat to Our Economic Future (Discussion Draft).

[2] O’Neill J. (2016). Review on Antimicrobial Resistance. Tackling drug-resistant infections globally: Final report and recommendations. Tackling Drug-Resistant Infections Globally.

[3] Australian Commission on Safety and Quality in Health Care AURA 2023: Fifth Australian Report on Antimicrobial Use and Resistance in Human Health. https://www.safetyandquality.gov.au/our-work/antimicrobial-resistance/antimicrobial-use-and-resistance-australia-aura/aura-2023-fifth-australian-report-antimicrobial-use-and-resistance-human-health.

[4] Datta R., Kiwak E., Fried T.R., Benjamin A., Iannone L., Krein S.L., Cohen A.B. (2024). Diagnostic uncertainty and decision-making in home-based primary care: A qualitative study. J. Am. Geriatr. Soc..

[5] Kasse G.E., Humphries J., Cosh S.M., Islam M.S. (2024). Factors contributing to the variation in antibiotic prescribing among primary health care physicians: A systematic review. BMC Prim. Care.

[6] Charton L., Severac F., Hansmann Y., Chambe J. (2025). Factors influencing inappropriate antibiotic prescription in respiratory tract infections in general practice. Sci. Rep..

[7] Wharton A., Jerome-D’Emilia B., Avallone M. (2024). Improving antibiotic overuse in primary care: A multimodal quality improvement project. Clin. Nurse Spec..

[8] Cziner M.J., Park D.E., Hamdy R.F., Rogers L.A., Turner M.M., Liu C.M. (2023). Effects of patient beliefs regarding the need for antibiotics and prescribing outcomes on patient satisfaction in urgent-care settings. Antimicrob. Steward. Healthc. Epidemiol..

[9] Simeoni M., Saragosa M., Laur C., Desveaux L., Schwartz K., Ivers N. (2022). Coping with “the grey area” of antibiotic prescribing: A theory-informed qualitative study exploring family physician perspectives on antibiotic prescribing. BMC Prim. Care.

[10] Ashworth M., White P., Jongsma H., Schofield P., Armstrong D. (2016). Antibiotic prescribing and patient satisfaction in primary care in England: Cross-sectional analysis of national patient survey data and prescribing data. Br. J. Gen. Pract..

[11] Biezen R., Brijnath B., Grando D., Mazza D. (2017). Management of respiratory tract infections in young children: A qualitative study of primary care providers’ perspectives. NPJ Prim. Care Respir. Med..

[12] Australian Commission on Safety and Quality in Health Care (2025). Antimicrobial Stewardship Clinical Care Standard.

[13] Australian Commission on Safety and Quality in Health Care Decision Support Tools. https://www.safetyandquality.gov.au/our-work/shared-decision-making/patient-decision-aids/.

[14] Biezen R., Ciavarella S., Manski-Nankervis J.-A., Monaghan T., Buising K. (2023). Addressing antimicrobial stewardship in primary care: Developing patient information sheets using co-design methodology. Antibiotics.

[15] National Centre for Antimicrobial Stewardship (2020). Community Information Sheets.

[16] Bakhit M., Del Mar C., Gibson E., Hoffmann T. (2018). Shared decision making and antibiotic benefit–harm conversations: An observational study of consultations between general practitioners and patients with acute respiratory infections. BMC Fam. Pract..

[17] Del Mar C., Hoffmann T., Bakhit M. (2022). How can general practitioners reduce antibiotic prescribing in collaboration with their patients?. Aust. J. Gen. Pract..

[18] van Horrik T.M.Z.X.K., Laan B.J., van Seben R., Rodenburg G., Heeregrave E.J., Geerlings S.E. (2022). Shared decision making for women with uncomplicated cystitis in primary care in the Netherlands: A qualitative interview study. BMC Prim. Care.

[19] van der Zande M.M., Dembinsky M., Aresi G., van Staa T.P. (2019). General practitioners’ accounts of negotiating antibiotic prescribing decisions with patients: A qualitative study on what influences antibiotic prescribing in low, medium and high prescribing practices. BMC Fam. Pract..

[20] Coronado-Vázquez V., Canet-Fajas C., Delgado-Marroquín M.T., Magallón-Botaya R., Romero-Martín M., Gómez-Salgado J. (2020). Interventions to facilitate shared decision making using decision aids with patients in primary health care: A systematic review. Medicine.

[21] Wang D., Liu C., Wang X., Zhang X. (2020). Association between physicians’ perception of shared decision making and antibiotic prescribing behavior in primary care in Hubei, China: A cross-sectional study. Antibiotics.

[22] van Esch T.E.M., Brabers A.E.M., Hek K., van Dijk L., Verheij R.A., de Jong J.D. (2018). Does shared decision making reduce antibiotic prescribing in primary care?. J. Antimicrob. Chemother..

[23] Tu K., Sarkadi Kristiansson R., Gronsbell J., de Lusignan S., Flottorp S., Goh L.H., Hallinan C.M., Hoang U., Kang S.Y., Kim Y.S. (2022). Changes in primary care visits arising from the COVID-19 pandemic: An international comparative study by the International Consortium of Primary Care Big Data Researchers (INTRePID). BMJ Open.

