# Supplementary material for: Implementing Information Resources to Support Shared Decisions in Australian Primary Care: A Qualitative Perspective of an Antimicrobial Stewardship Strategy
Source: Antibiotics (Basel). 2026 Feb 17;15(2):216. doi: 10.3390/antibiotics15020216 (PMC12937397; doi:10.3390/antibiotics15020216)
Supplement: Supplementary file 1 [file antibiotics-15-00216-s001.zip › Table S1 Characteristics of primary care providers.pdf]

Table S1. Characteristics of primary care providers

Primary care provider ID	Gender	Age range (years)	Location of practice	Work fraction	Years of experience in general practice
GP1	Male	41-50	Regional/rural	Full-time	10
GP2	Male	31-40	Regional/rural	Full-time	6
GP3	Male	61-70	Metropolitan	Full-time	38
GP4	Female	51-60	Metropolitan	Part-time	31
GP5	Male	41-50	Regional/rural	Full-time	12
GP6	Male	31-40	Regional/rural	Full-time	4.5
GP7	Male	41-50	Metropolitan	Full-time	20
GP9	Female	41-50	Metropolitan	Full-time	4
GP10	Female	61-70	Metropolitan	Part-time	40
GP11	Female	41-50	Regional/rural	Part-time	21
GP12	Female	31-40	Regional/rural	Full-time	1
GP13	Female	51-60	Metropolitan	Part-time	30
GP14	Male	31-40	Regional/rural	Part-time	1
GP15	Female	31-40	Regional/rural	Part-time	2
PN1	Female	25-30	Metropolitan	Part-time	6

(GP8 did not participate in the interview).
