# Supplementary material for: Implementing Information Resources to Support Shared Decisions in Australian Primary Care: A Qualitative Perspective of an Antimicrobial Stewardship Strategy
Source: Antibiotics (Basel). 2026 Feb 17;15(2):216. doi: 10.3390/antibiotics15020216 (PMC12937397; doi:10.3390/antibiotics15020216)
Supplement: Supplementary file 1 [file antibiotics-15-00216-s001.zip › Table S2 Characteristics of patients.pdf]

Table S2. Characteristics of patients

Patient ID	Gender	Age range (years)	Residential location	Highest qualification
Patient 1	Female	46-55	Regional/rural	University degree
Patient 2	Female	36-45	Regional/rural	Year 12
Patient 3	Male	18-25	Regional/rural	Trade Certificate
Patient 4	Female	46-55	Regional/rural	Trade certificate
Patient 5	Male	36-45	Metropolitan	University degree
Patient 6	Male	65+	Metropolitan	Trade certificate
Patient 7	Female	65+	Metropolitan	Post graduate degree
Patient 8	Female	65+	Metropolitan	Year 9
Patient 9	Female	56-65	Metropolitan	Year 10
Patient 10	Male	56-65	Metropolitan	Trade certificate
Patient 11	Female	46-55	Metropolitan	Post graduate degree
Patient 12	Male	65+	Metropolitan	Post graduate degree
Patient 13	Female	26-35	Metropolitan	Post graduate degree
